# The Polybasic Cleavage Site in SARS-CoV-2 Spike Modulates Viral Sensitivity to Type I Interferon and IFITM2

**DOI:** 10.1128/JVI.02422-20

**Published:** 2021-04-12

**Authors:** Helena Winstone, Maria Jose Lista, Alisha C. Reid, Clement Bouton, Suzanne Pickering, Rui Pedro Galao, Claire Kerridge, Katie J. Doores, Chad M. Swanson, Stuart J. D. Neil

**Affiliations:** a Department of Infectious Diseases, School of Immunology and Microbial Sciences, King’s College London, London, United Kingdom; Loyola University Chicago

**Keywords:** furin cleavage, IFITM2, innate immunity, SARS-CoV-2, spike, type 1 interferon

## Abstract

The cellular entry of severe acute respiratory syndrome-associated coronaviruses types 1 and 2 (SARS-CoV-1 and -2) requires sequential protease processing of the viral spike glycoprotein. The presence of a polybasic cleavage site in SARS-CoV-2 spike at the S1/S2 boundary has been suggested to be a factor in the increased transmissibility of SARS-CoV-2 compared to SARS-CoV-1 by facilitating maturation of the spike precursor by furin-like proteases in the producer cells rather than endosomal cathepsins in the target. We investigate the relevance of the polybasic cleavage site in the route of entry of SARS-CoV-2 and the consequences this has for sensitivity to interferons (IFNs) and, more specifically, the IFN-induced transmembrane (IFITM) protein family that inhibit entry of diverse enveloped viruses. We found that SARS-CoV-2 is restricted predominantly by IFITM2, rather than IFITM3, and the degree of this restriction is governed by route of viral entry. Importantly, removal of the cleavage site in the spike protein renders SARS-CoV-2 entry highly pH and cathepsin dependent in late endosomes, where, like SARS-CoV-1 spike, it is more sensitive to IFITM2 restriction. Furthermore, we found that potent inhibition of SARS-CoV-2 replication by type I but not type II IFNs is alleviated by targeted depletion of IFITM2 expression. We propose that the polybasic cleavage site allows SARS-CoV-2 to mediate viral entry in a pH-independent manner, in part to mitigate against IFITM-mediated restriction and promote replication and transmission. This suggests that therapeutic strategies that target furin-mediated cleavage of SARS-CoV-2 spike may reduce viral replication through the activity of type I IFNs.

**IMPORTANCE** The furin cleavage site in the spike protein is a distinguishing feature of SARS-CoV-2 and has been proposed to be a determinant for the higher transmissibility between individuals, compared to SARS-CoV-1. One explanation for this is that it permits more efficient activation of fusion at or near the cell surface rather than requiring processing in the endosome of the target cell. Here, we show that SARS-CoV-2 is inhibited by antiviral membrane protein IFITM2 and that the sensitivity is exacerbated by deletion of the furin cleavage site, which restricts viral entry to low pH compartments. Furthermore, we find that IFITM2 is a significant effector of the antiviral activity of type I interferons against SARS-CoV-2 replication. We suggest that one role of the furin cleavage site is to reduce SARS-CoV-2 sensitivity to innate immune restriction, and thus, it may represent a potential therapeutic target for COVID-19 treatment development.

## Abstract (precis)

The furin cleavage site in the spike protein is a distinguishing feature of SARS-CoV-2 and has been proposed to be a determinant for the higher transmissibility between individuals, compared to SARS-CoV-1. One explanation for this is that it permits more efficient activation of fusion at or near the cell surface rather than requiring processing in the endosome of the target cell.

## INTRODUCTION

Severe acute respiratory syndrome coronavirus 2 (SARS-CoV-2) is a novel coronavirus that was identified in early 2020 (1). Entry of SARS-CoV-2 into the target cell is initiated by the spike glycoprotein binding to its receptor, angiotensin-converting enzyme 2 (ACE2) (2). Spike is a type I transmembrane protein that is synthesized as a polyprotein precursor and requires two steps of proteolytic cleavage at the S1/S2 boundary and at the S2′ site in order to mediate fusion of the viral and cell membranes. Due to the insertion of four amino acids (in bold) at the S1/S2 boundary of SARS-CoV-2 spike, with the sequence _681_**PRRA**R/SV_687_, SARS-CoV-2 spike contains a canonical furin-like protease cleavage site (2). This allows the SARS-CoV-2 spike to be cleaved by furin-like proteases intracellularly prior to virion release. TMPRSS2 on the target cell surface and cathepsins B and L in endosomes may then cleave the S2′ site and activate the fusion machinery, depending on the relative availability of these enzymes.

The presence of the furin cleavage site has been suggested to be important for determining viral tropism and transmission of SARS-CoV-2 (3–5). However, the necessity for this site is cell type dependent. It has been shown that this site can be lost after several passages in TMPRSS2-negative Vero E6 cells (6). Nevertheless, similar mutations have only been found rarely in a small number of patients (7, 8). This suggests a selective pressure to conserve the polybasic cleavage site for *in vivo* transmission but not necessarily *in vitro*, depending on the cell line used (5–8). Structural data for SARS-CoV-2 spike indicates that cleavage at the S1/S2 boundary results in exposure of the receptor-binding domain (RBD) of spike (9). It has been suggested that this exposure of the RBD facilitates binding to ACE2 and the secondary cleavage of the S2′ site of spike, facilitating membrane fusion.

Interferons (IFNs) upregulate the expression of a range of antiviral proteins, encoded by genes termed IFN-stimulated genes (ISGs), that inhibit various aspects of viral life cycles, including entry (10). One of these protein families, IFN-induced transmembrane proteins (IFITMs), are membrane-spanning proteins that inhibit the entry of several viruses, including HIV-1, influenza virus, Ebola virus, and SARS-CoV-1, through blocking the fusion of the cellular and viral membranes, possibly by decreasing membrane fluidity or affecting membrane curvature (11, 12). Three IFITMs demonstrate antiviral activity in humans: IFITM1, which localizes to the plasma membrane, and IFITM2 and -3, which localize to late and early endosomes, respectively (13, 14). Previous research has shown that the route of entry correlates with the restriction of both influenza virus and HIV-1 by IFITMs. Mislocalizing IFITM3 to the cell surface abrogates IFITM3 restriction of influenza virus (15). CCR5-tropic HIV-1 viruses that fuse at the plasma membrane are more restricted by IFITM1, while CXCR4-tropic viruses that utilize the endosomal route are more restricted by IFITM2 and -3 (13). It has been reported that SARS-CoV-2 is highly sensitive to type I and III IFNs and, more specifically, to IFITM3 (5, 16–18). Conversely, other authors have suggested that expression of IFITMs can enhance entry of SARS-CoV-2 (19). Given that entry is the first key step in viral transmission and IFITMs have been shown to be expressed in lung tissue, the interplay between IFITM restriction and the route of SARS-CoV-2 entry is likely to be fundamental to the ability of SARS-CoV-2 to infect and to be transmitted (20, 21). Here, we show the differential sensitivity of SARS-CoV-2 to IFITMs and how the presence of a polybasic cleavage site may affect entry in the context of IFITM restriction.

## RESULTS

### Sensitivity of SARS-CoV-2 and pseudotyped lentiviral vectors (PLVs) with SARS-CoV-2 spike to human type I, type II, and type III interferons in A549-ACE2 cells.

**FIG 1:**
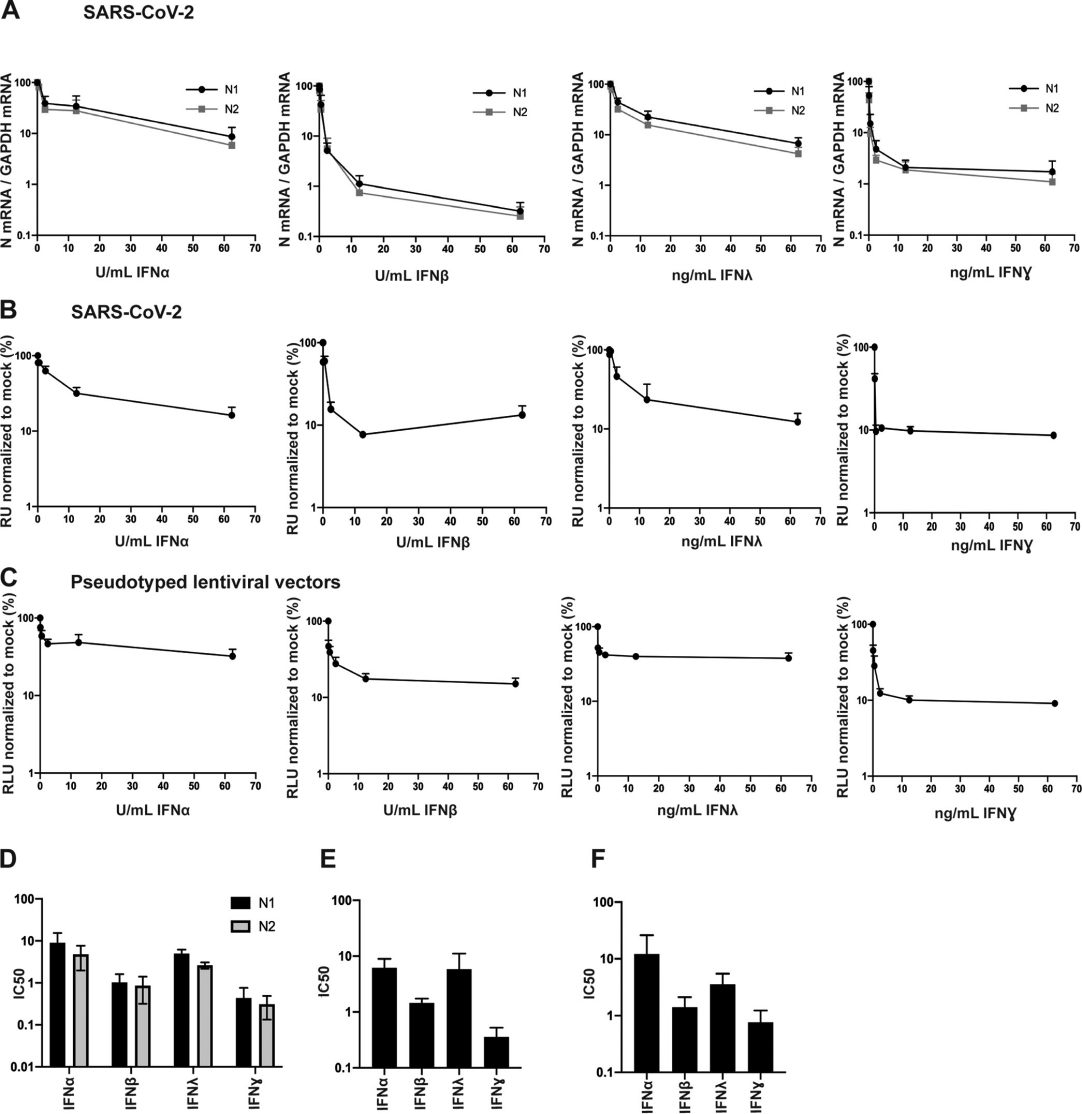
Entry of replication-competent SARS-CoV-2 and PLVs of SARS-CoV-2 is inhibited by IFN-β and IFN-γ. (A) A549-ACE2 cells were pretreated for 18 h with IFN-α, -β, -λ, or -γ and subsequently infected with replication-competent SARS-CoV-2 at an MOI of 0.005. Viral RNA was extracted 48 h later, detected with two sets of primers (N1 and N2) against nucleocapsid mRNA, and normalized to infection in mock-treated cells. (B) Supernatant from infected A549-ACE2 used for panel A was used to infect Vero E6 cells for 24 h. Vero E6 cells were then stained for nucleocapsid protein and normalized to mock-treated conditions. RU, relative units. (C) A549-ACE2 cells were pretreated for 18 h with IFN-α, -β, -λ, or -γ and transduced with PLVs of SARS-CoV-2 for 48 h. Infection was quantified by luciferase activity and normalized to mock-treated conditions. RLU, relative luminescence units. (D to F) IC_50_s for panels A to C were calculated in Prism. All data are means and standard errors of the means (SEM) (*n* = 3).

In order to examine the restriction of SARS-CoV-2 replication by human antiviral proteins, we first sought to confirm the sensitivity of replication-competent SARS-CoV-2 (SARS-CoV-2 strain England 2) to type I (α and β), type II (γ), and type III (λ) IFNs in human A549 lung cancer cells stably expressing ACE2. We pretreated the cells with different doses of recombinant human IFN-α2, -β, -λ4, and -γ overnight and then challenged them with SARS-CoV-2 (multiplicity of infection [MOI] of 0.005 based on Vero E6 cells). Then, 48 h later we measured the levels of viral RNA by reverse transcription-quantitative PCR (RT-qPCR) using the N1 and N2 primer probe sets from Centers for Disease Control N1 and N2 primer probe sets (Fig. 1A and D). We found that SARS-CoV-2 is highly sensitive to IFN-β and IFN-γ, with very low half-maximal inhibitory concentrations (IC_50_s), and is less sensitive but nonetheless still restricted by IFN-α and IFN-λ. In addition to measuring intracellular viral RNA abundance in the IFN-treated cells, we infected Vero E6 cells with the supernatant harvested from IFN-treated and infected A549-ACE2 cells and quantified the expression of nucleocapsid (N) protein 24 h later. This assay measures the amount of infectious virus produced by the mock or IFN-treated cells and showed similar results (Fig. 1B and E), thus confirming previous studies showing that the virus is highly IFN sensitive, particularly to IFN-β and IFN-γ, and indicating that a number of ISGs have direct antiviral effects against SARS-CoV-2 (16, 18).

In order to address the activities of ISGs directed against spike-mediated entry, we first determined whether we could recapitulate the IFN phenotypes observed above using pseudotyped lentiviral vectors (PLVs). We generated PLVs containing SARS-CoV-2 spike bearing a luciferase reporter gene and tested them for sensitivity to IFNs on A549-ACE2. Similar to full-length SARS-CoV-2, we found that PLVs with SARS-CoV-2 spike are also highly sensitive to IFN-β and IFN-γ (Fig. 1C and F). While the early events of HIV-1 are known targets of IFN treatment in some cell lines, these data suggest that when we isolated the entry stage of SARS-CoV-2 infection, we observed inhibition by IFN-β and IFN-γ (22).

### SARS-CoV-2 is sensitive to IFITM2, but not IFITM3, in A549-ACE2 cells.

IFITMs are a family of ISGs that predominantly inhibit fusion of viral and cellular membranes (11, 14). Considering that our PLVs with SARS-CoV-2 spike demonstrated an extent of inhibition by IFNs similar to that of the full-length virus, we suspected that IFITMs, which have previously been reported to inhibit SARS-CoV-1 and more recently suggested to inhibit SARS-CoV-2, may contribute to this inhibition (5, 12, 19, 23).

**FIG 2:**
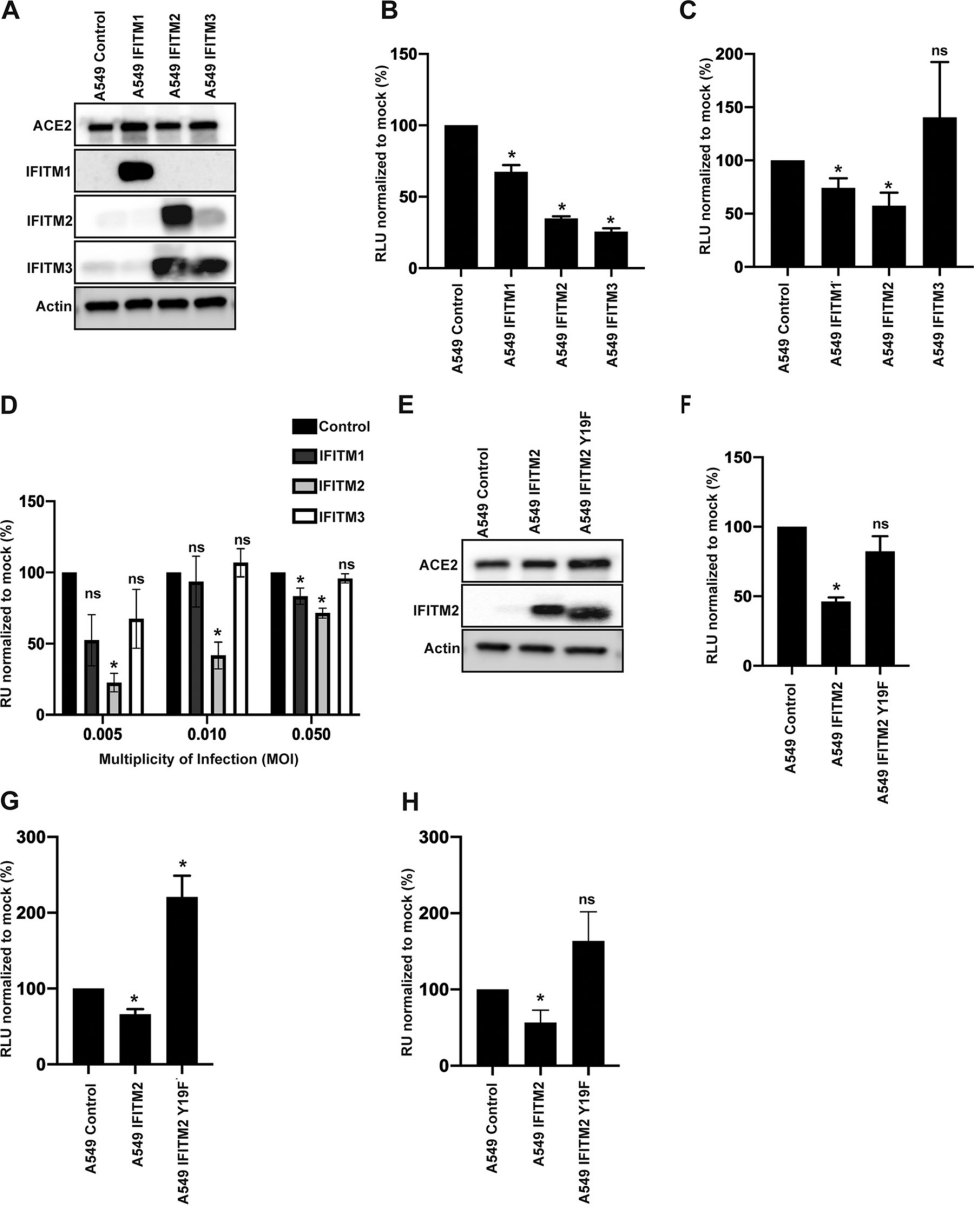
Replication-competent SARS-CoV-2 and PLVs of SARS-CoV-2 are inhibited by IFITM2 in A549-ACE2 cells. (A) Representative immunoblot of A549-ACE2 cells stably expressing IFITM1, IFITM2, and IFITM3. Of note, the antibody to IFITM2 and IFITM3 recognizes both proteins. (B) A549-ACE2-IFITM cells were infected with influenza A virus (IAV), and infection was quantified by luciferase activity 24 h later. (C) A549-ACE2-IFITM cells were transduced with SARS-CoV-2 PLVs for 48 h, and infection was quantified by luciferase activity. (D) A549-ACE2-IFITM cells were infected with replication-competent SARS-CoV-2 for 48 h at MOI of 0.005, 0.01, and 0.05. Supernatant was then used to infect Vero E6 cells for 24 h, and cells were stained for nucleocapsid protein. (E) Representative immunoblot of A549-ACE2 cells stably expressing IFITM2 and IFITM2 Y19F. (F) A549-ACE2 IFITM cells used for panel E were infected with IAV, and infection was quantified by luciferase activity 24 h later. (G) SARS-CoV-2 PLVs were used to transduce A549-ACE2-IFITM2 and the Y19F mutant, and infection was quantified 48 h later by luciferase activity. (H) Replication-competent SARS-CoV-2 was used to infect A549-ACE2-IFITM2 cells or A549-ACE2 cells stably expressing IFITM2 or IFITM2 Y19F at an MOI of 0.005. Supernatant was used to infect Vero E6 cells for 24 h, and cells were N stained as for panel C. RU, relative units; RLU, relative luminescence units. All data are means and SEM (*n* = 3). *, *P* < 0.05 (unpaired *t* test, calculated in Prism).

To test the impact of each individual IFITM on SARS-CoV-2 infection, we generated stable A549-ACE2 cell lines expressing each human antiviral IFITM (Fig. 2A). Of note, cross-reactivity between antibodies targeting IFITM2 and IFITM3 is inevitable due to high homology between these proteins. We infected these cells with influenza A virus (IAV) and confirmed that, consistent with previous findings, overexpression of IFITM2 and IFITM3 inhibited IAV infection (Fig. 2B) (24, 25). Next, we infected these cells with PLVs and found that SARS-CoV-2 showed a small but significant sensitivity to IFITM1 and a greater sensitivity to IFITM2 (Fig. 2C). We recapitulated these phenotypes by challenging the A549-ACE2-IFITM cells with SARS-CoV-2 at MOI of 0.005, 0.01, and 0.05. We used the supernatants from these cells 48 h later to infect Vero E6 cells and measured viral infectivity by staining for N protein (Fig. 2D). At low MOI, SARS-CoV-2 was particularly sensitive to IFITM2 but not IFITM3, with an inhibitory effect seen with IFITM1, and these sensitivities were ameliorated at high viral inputs. As both single-round PLVs and the full-length virus essentially displayed similar phenotypic sensitivity to IFITM2, these results suggest that a predominant antiviral effect is mediated at cellular entry.

Both IFITM2 and IFITM3 predominantly localize to endosomal compartments but reach them via endocytosis from the cell surface, through the recruitment of the clathrin adaptor AP2 to a tyrosine-based endocytic signal (YXXΦ) in the IFITM2/3 cytoplasmic tail. We and others have previously demonstrated that mutating Y19 and Y20 to a phenylalanine in IFITM2 and IFITM3, respectively, results in their accumulation at the plasma membrane (13, 26). To test this, we stably expressed IFITM2 Y19F A549-ACE2 (Fig. 2E) and infected these cells with IAV (Fig. 2F). We found that infection of IFITM2-Y19F cells was slightly enhanced compared to that of IFITM2 cells. Similarly, infection of these cells with PLVs and replication-competent SARS-CoV-2 at an MOI of 0.005 revealed that infection was not inhibited but rather was enhanced by the presence of IFITM2-Y19F (Fig. 2G and H). Although it was surprising that mislocalization of IFITM2 resulted in enhancement of infection rather than simply an absence of restriction, these data are consistent with a recent report suggesting that similar mutants of IFITM3 enhance SARS-CoV-2 infection (27). These data suggest that the localization of IFITM2 to endosomes or its recruitment to clathrin-coated pits at the plasma membrane is key to its inhibition of SARS-CoV-2 entry.

### The polybasic cleavage site determines sensitivity to IFITM2 in the presence or absence of TMPRSS2.

**FIG 3:**
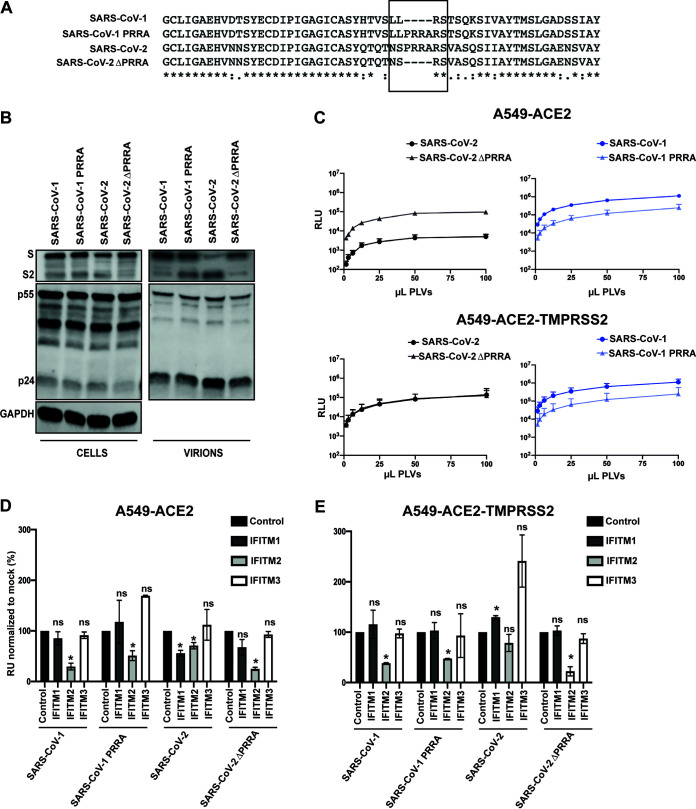
The presence or absence of a polybasic cleavage site determines sensitivity to IFITM2. (A) Alignment of the S1/S2 boundary in SARS-CoV-1 and SARS-CoV-2 with mutants where PRRA has been inserted/deleted. Alignment was created in Clustal Omega. (B) Representative immunoblot of PLVs of SARS-CoV-1, SARS-CoV-1 PRRA, SARS-CoV-2, and SARS-CoV-2ΔPRRA. (C) PLVs of SARS-CoV-1, SARS-CoV-1 PRRA, SARS-CoV-2, and SARS-CoV-2ΔPRRA were titrated on A549-ACE2 or A549-ACE2-TMPRSS2 cells, and infectivity was measured by luciferase assay 48 h later. (D and E) PLVs of SARS, as described for panels B and C, were used to transduce A549-ACE2-IFITM cells (D) or A549-ACE2-TMPRSS2-IFITM cells (E) for 48 h, and infection was measured by luciferase activity. Infection was normalized to empty vector cells. RLU, relative luminescence units. All data are means and SEM (*n* = 3). *, *P* < 0.05 (unpaired *t* test, calculated in Prism).

A major difference between the spike protein of SARS-CoV-2 and that of the majority of the phylogenetically related bat sarbecoviruses, including SARS-CoV-1, is the presence of the polybasic cleavage site at the S1/S2 boundary (Fig. 3A). This facilitates the processing of spike to S1/S2 during viral assembly in the producer cell rather than during entry into the target cell. As this feature has been proposed to be associated with the increased transmissibility of SARS-CoV-2, we hypothesized that it might affect the sensitivity of the virus to IFITM2. To investigate this, we deleted the polybasic cleavage site from SARS-CoV-2 (while preserving the adjacent RS serine protease cleavage site) and swapped the corresponding region from (P_681_-A_684_) SARS-CoV-2 into SARS-CoV-1, generating SARS-CoV-2ΔPRRA and SARS-CoV-1 PRRA, respectively (Fig. 3A). We made PLVs of these mutants and analyzed spike expression and virion incorporation by Western blotting using a polyclonal antibody against SARS-CoV-1/2 S2 (Fig. 3B). We found that all spike proteins were equivalently expressed in the transfected producer 293T-17 cells. As expected, the SARS-CoV-1 spike existed predominantly as the S1/2 precursor on pelleted virions in the supernatant. In contrast, processed S2 was the predominant species found on virions pseudotyped with SARS-CoV-2 spike, indicating furin-mediated cleavage during virion assembly. As expected, SARS-CoV-2ΔPRRA was not cleaved. Insertion of the SARS-CoV-2 cleavage site into SARS-CoV-1 was sufficient to lead to processed spike; however, this was not as efficient as in SARS-CoV-2, with virions incorporating both cleaved and uncleaved spike (Fig. 3B). In keeping with results from others in Vero E6 cells, in A549 cells SARS-CoV-2ΔPRRA PLVs had a marked increase in infectivity of approximately 50-fold in A549-ACE2 cells compared to the wild-type spike, approaching the infectivity of SARS-CoV-1 (Fig. 3C) (4). Addition of the PRRA site to SARS-CoV-1 slightly reduced titers. Since SARS-CoV-2 requires TMPRSS2 in the target cells to activate spike for entry, we overexpressed TMPRSS2 in A549-ACE2 cells by retroviral transduction. This specifically enhanced infection of SARS-CoV-2 PLVs, indicating that, in the absence of TMPRSS2 expression, much of the SARS-CoV-2 inoculum is not infectious in these cells.

We then tested IFITM sensitivity of these PLVs in A549-ACE2 cells with and without TMPRSS2 overexpression (Fig. 3D and E). As expected, SARS-CoV-2 PLVs were sensitive to both IFITM1 and IFITM2 in A549-ACE2 cells (Fig. 3D). SARS-CoV-1 PLVs were significantly more sensitive to IFITM2 but displayed no restriction by IFITM1, suggestive of distinct subcellular site of entry between SARS-CoV-1 and SARS-CoV-2. Interestingly, deletion of PRRA in SARS-CoV-2 rendered this spike as sensitive as SARS-CoV-1 to IFITM2 and slightly reduced the IFITM1 sensitivity. In contrast, the addition of a cleavage site to SARS-CoV-1 significantly reduced IFITM2 sensitivity, albeit not to the levels of the fully cleaved SARS-CoV-2 spike. When we overexpressed TMPRSS2, we found that while IFITM1 sensitivity of SARS-CoV-2 could be abolished, this was not sufficient to rescue SARS-CoV-2 or SARS-CoV-2ΔPRRA from IFITM2 (Fig. 3D). Thus, the presence of the polybasic cleavage site markedly reduces the sensitivity of SARS-CoV-2 S-mediated entry to IFITM2, suggesting that it affects the route of entry into the cell and distinguishes this virus from SARS-CoV-1.

**FIG 4:**
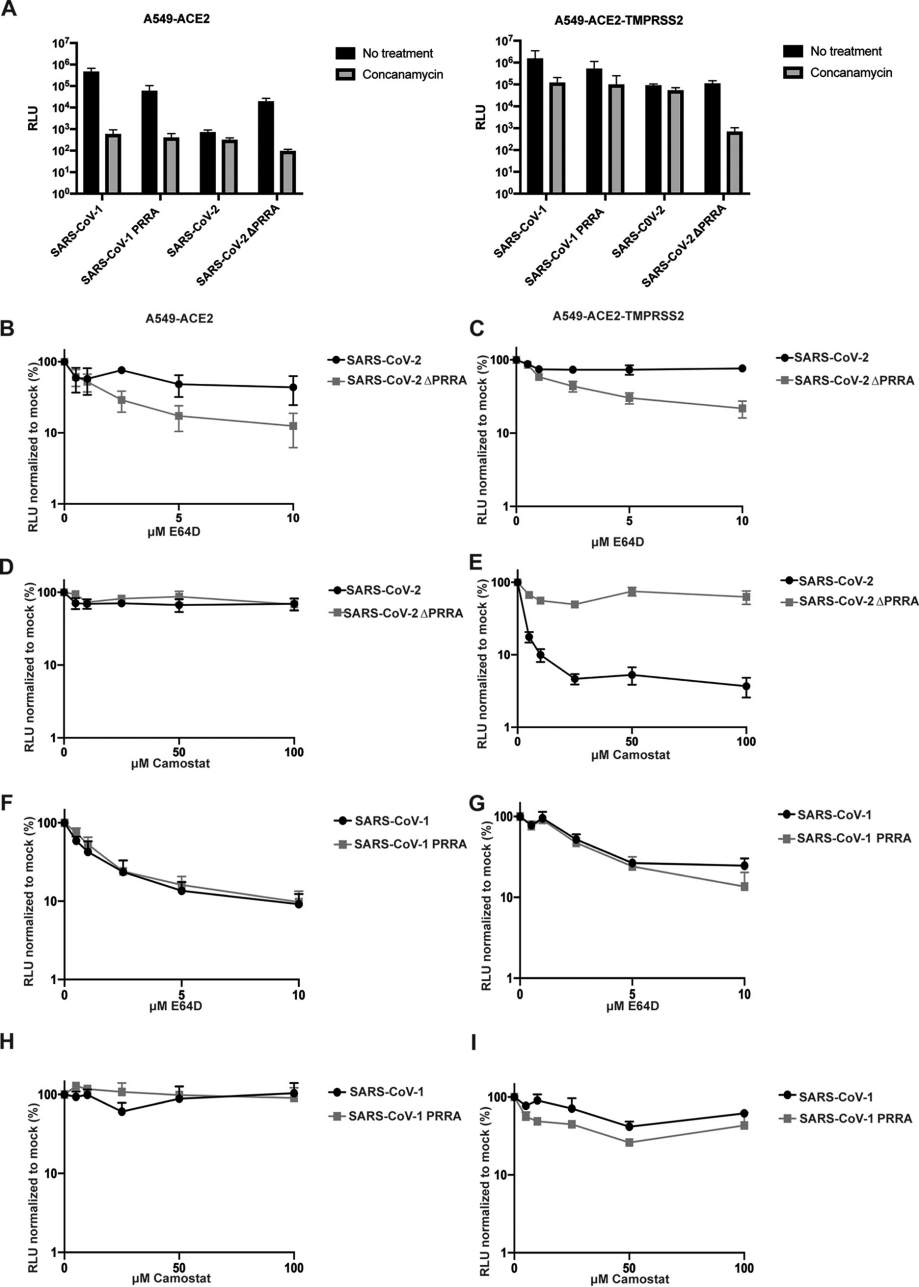
SARS-CoV-2 and SARS-CoV-2ΔPRRA differ in preferential route of entry. (A) A549-ACE2 and A549-ACE2-TMPRSS2 cells were treated with 100 nM concanamycin for 1 h and transduced with SARS PLVs. Infection was determined by luciferase activity 48 h later. Black, nontreated; gray, concanamycin. (B to E) A549-ACE2 and A549-ACE2-TMPRSS2 cells were treated for 1 h with E64d or camostat and subsequently transduced with PLVs of SARS-CoV-2 or SARS-CoV-2ΔPRRA, and infection was detected by luciferase activity 48 h later. (F to I) A549-ACE2 or A549-ACE2-TMPRSS2 cells were treated with E64d or camostat for 1 h and transduced with PLVs of SARS-CoV-1 or SARS-CoV-1 PRRA, and infection was detected by luciferase activity 48 h later. RLU, relative luminescence units. All data are means and SEM (*n* = 3).

To address the effects of spike cleavage on route of entry, we first determined the pH sensitivity of the spike cleavage mutants using concanamycin A (ConA), an inhibitor of the vacuolar ATPase in late endosomes (Fig. 4A). As expected, SARS-CoV-1 PLVs were exquisitely sensitive (1,000-fold) to ConA inhibition in A549-ACE2, indicating that entry occurred exclusively in a low-pH endosomal compartment. In the presence of TMPRSS2, SARS-CoV-1 pH sensitivity was reduced, but entry still remained 20- to 50-fold lower, suggesting that any enhanced S2′ processing was not sufficient to abolish pH-dependent entry. Similarly, while insertion of a partially processed polybasic cleavage site in SARS-CoV-1 reduced but did not abolish pH-dependent entry in either cell type. In contrast, entry of SARS CoV-2 PLVs was only mildly affected (2- to 3-fold) by ConA treatment irrespective of TMPRSS2 overexpression, indicating that most viral entry occurred at neutral pH and that TMPRSS2 enhanced entry at this point rather than elsewhere in the cell. Similar to SARS-CoV-1, deletion of the PRRA site from SARS-CoV-2 rendered PLVs strictly pH dependent without affecting titers. In keeping with these data, unlike SARS-CoV-2-, SARS-CoV-1-, and SARS-CoV-2ΔPRRA-mediated entry was inhibited by the endosomal cathepsin inhibitor E64D but not the TMPRSS inhibitor camostat (Fig. 4B to I). In contrast, SARS-CoV-2 was sensitive to E64D only in TMPRSS2-overexpressing cells. Together, these data suggest that S1/S2 cleavage by furin in the producer cell promotes TMPRSS2-mediated entry at the plasma membrane, or soon after internalization, and abolishes the requirement for cathepsin-mediated processing in the acidic endosomal compartments. The data further suggest that in the absence of abundant TMPRSS2 at the cell surface, the processed SARS-CoV-2 cannot efficiently enter through a low-pH compartment. Thus, the PRRA site dictates the route of entry into the cell and therefore its sensitivity to IFITM proteins that occupy different cellular locations.

### IFITM2 contributes to the antiviral restriction of SARS-CoV-2 by IFN-β.

**FIG 5:**
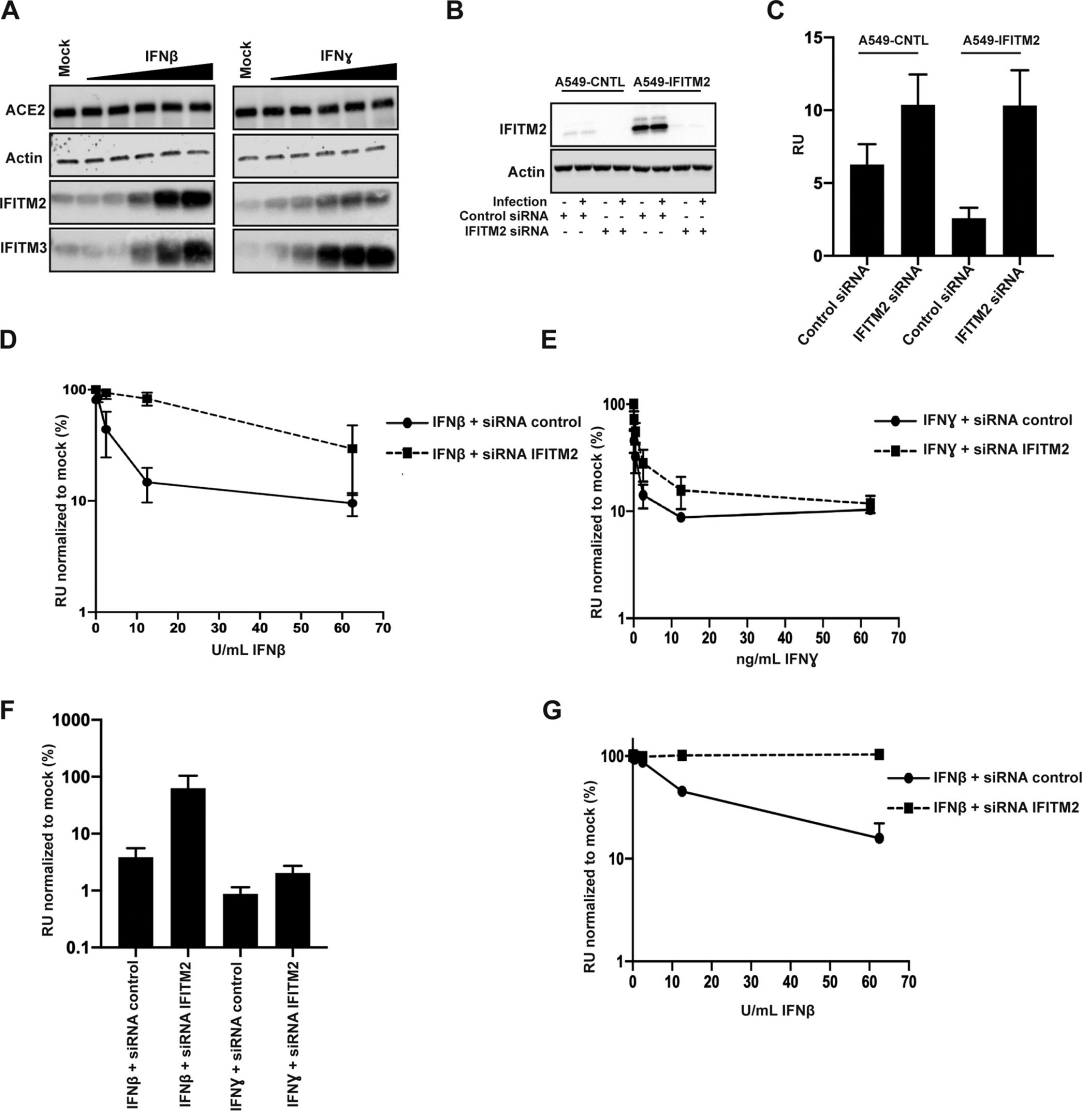
siRNA of IFITM2 rescues IFN-β-mediated restriction of replication competent SARS-CoV-2. (A) Representative immunoblot of A549-ACE2 treated with different amounts of IFN-β or IFN-γ for 18 h. (B and C) A549-ACE2 cells were transfected with siRNAs against nontargeting control or IFITM2; supernatants were used to infect Vero E6 cells for 24 h, and cells were stained for nucleocapsid protein. (D and E) A549-ACE2 cells were pretreated with IFN-β and IFN-γ for 18 h and infected with replication-competent SARS-CoV-2 at an MOI of 0.005. Infected supernatant was used to infect Vero E6 cells for 24 h, and cells were stained for N protein. (F) IC_50_s for panels D and E were calculated in Prism. (G) A549-ACE2-TMPRSS2 were transfected with siRNAs against nontargeting control or IFITM2 when seeding and prior to IFN treatment. Cells were treated with IFN-β and infected with replication-competent SARS-CoV-2 at an MOI of 0.005 18 h later. Infected supernatant was used to infect Vero E6 cells for 24 h, and cells were stained for N protein. RU, relative units. All data are means and SEM (*n* = 3).

Having established that IFITM2 can restrict SARS-CoV-2 depending on its mechanism of entry, we wanted to determine how much of the inhibition of replication-competent SARS-CoV-2 by IFN-β and IFN-γ could be attributed to IFITM2. We examined the expression of IFITM2 and IFITM3 in IFN-treated A549-ACE2 and observed a robust upregulation of both IFITM2 and IFITM3 following treatment with IFN-β. In contrast, while IFITM3 was also robustly induced by IFN-γ, IFITM2 was weakly induced (Fig. 5A). Using small interfering RNAs (siRNAs) against IFITM2 that rescued SARS-CoV-2 replication in A549-ACE2-IFITM2 cells (Fig. 5B and C), we then knocked down IFITM2 in the context of pretreating A549-ACE2 cells with IFN-β or IFN-γ and challenged the cells with SARS-CoV-2, measuring infectious virus output on Vero E6 cells 48 h later (Fig. 5D and E). IFITM2 depletion substantially relieved the inhibition of viral replication by IFN-β treatment, whereas that induced by IFN-γ was only modestly alleviated. This was reflected in the 20-fold increase in the IC_50_ of IFN-β, but only a 2-fold increase in IFN-γ, indicating that in these cells IFITM2 is a major component of the type I IFN-antiviral state protecting cells from SARS-CoV-2 (Fig. 5F). Furthermore, in A549-ACE2 cells overexpressing TMPRSS2, the knockdown of IFITM2 essentially abolished all the antiviral activity of pretreating the cells with IFN-β (Fig. 5G). Thus, IFITM2-mediated entry restriction is a major type I IFN activity that constitutes an antiviral state, blocking the replication of SARS CoV-2.

## DISCUSSION

In this study, we provide evidence that IFITM2 has potent inhibitory activity against SARS-CoV-2 entry and constitutes at least part of the antiviral activity conferred by treatment of target cells with IFN-β. Furthermore, we find that the presence or absence of the polybasic cleavage site, which facilitates pH-independent entry of SARS-CoV-2, modulates the sensitivity of the virus to IFITM2. In contrast to SARS-CoV-1 and other related SARS-like CoVs in bats, SARS-CoV-2 is distinguished by the presence of a furin cleavage site at the S1/S2 boundary (4). This leads to the spike on SARS-CoV-2 virions being predominantly cleaved in the producer cell rather than by cathepsins during endocytic entry into the target cell and renders its entry pH independent, suggesting that fusion occurs at, or near, the cell surface. Recent evidence further indicates that furin cleavage generates a C-terminal ligand on S1 that interacts with neuropilin-1 (NRP-1) on the surface of target cells in the lung (28, 29). The role of NRP-1 is not completely clear, but there is some suggestion that it may stabilize the attachment of SARS-CoV-2 at the cell surface to facilitate either ACE2 interaction or processing of the S2′ site by TMPRSS2. Structural analyses of the SARS-CoV-2 spike trimer further show that furin-mediated cleavage facilitates at least one RBD to adopt an erect conformation that would further promote ACE2 interaction (9). Interestingly, deletion of the PRRA is not detrimental to SARS-CoV-2 entry in all cell types in culture, and in TMPRSS2-low Vero E6 cells, the furin cleavage site is rapidly lost upon passage, suggesting that it can actively hinder infection (6). Herein, we show that while wild-type spike-mediated entry is insensitive to inhibition of endosomal pH, the cleavage mutant is strictly dependent on endosome acidification and cathepsins.

Interestingly, for efficient entry, SARS-CoV-2 requires high TMPRSS2 expression to activate the fusion mechanism by cleaving S2′. However, in cells where TMPRSS2 is limiting, SARS-CoV-1 and SARS-CoV-2ΔPRRA entry is far more efficient. Thus, in its uncleaved form, SARS-CoV-2 spike can mediate entry in endosomes, but in its mature form, entry cannot be rescued in low-pH compartments of TMPRSS2-low cells. This implies that the cleaved spike is unstable at endosomal pH, and, interestingly, recent studies from the Kwong group indicate that conformational dynamics of the RBD are also pH sensitive (30). Despite this potential greater fragility of the SARS-CoV-2 trimer, the furin cleavage site appears to be essential for replication in primary airway epithelium and for transmission in ferret models (5). We suggest that one of the reasons pH-independent fusion at or near the cell surface is maintained is to mitigate the antiviral activity of IFITM proteins, particularly IFITM2. We note that insertion of the PRRA site into SARS-CoV-1 does not result in cleavage of spike to the same extent as in SARS-CoV-2 or fully rescue sensitivity to IFITM2. We expect that this is due to other differences in the structure of these spikes besides the S1/S2 boundary, such as in the RBD.

The localization of IFITMs largely defines which viruses they restrict. While they can be incorporated into nascent virion membranes and exert an antiviral effect there, their best-studied mechanism of action is to prevent fusion of an incoming virus at the target cell membrane (14). IFITM1 is predominantly found at the plasma membrane, whereas IFITM2 and IFITM3 occupy endosomal compartments by virtue of a conserved endocytic signal. Palmitoylation of the intracellular loop of the IFITM stabilizes their conformation in the membrane and promotes their homo- and heterotypic interactions (31). The current model for their action is that IFITM-IFITM interactions exert a level of positive curvature to the target membrane that arrests enveloped viral entry at the hemi-fusion stage (32). IFITM3 is particularly potent against influenza viruses, and its redistribution away from early endosomes by mutating the endocytic site in the cytoplasmic tail abolishes its antiviral activity (24, 33). Less is known about IFITM2, although it has been shown to inhibit a number of other enveloped viruses that enter in later endosomes (13, 23). Of note, human IFITM2 and -3 differ from each other by only 10 amino acids, and yet their restriction patterns are not interchangeable. While IFITM2 and -3 are localized in endosomal compartments, they traffic via the cell surface, and their recruitment into clathrin-coated pits would imply that they may have some activity at viral entry sites at the plasma membrane as well. However, our observations that IFITM2, but not IFITM3, in the A549 system inhibits SARS-CoV-1 and -CoV-2 suggests that neither virus fuses significantly in a cellular compartment occupied by IFITM3 in this cell type. It is not yet clear which cell type most accurately models the interactions of SARS-CoV-2 and IFITMs in the lung; however, it is likely that the choice of cell and whether overexpression of IFITMs is transient or stable affect the pattern of IFITM restriction of SARS-CoV-2. Furthermore, cell type specificity in IFITM localization (due to endocytic rate, etc.) and heterotypic interactions between IFITMs suggest that when all are coexpressed, they may form a more complex barrier to enveloped virus fusion than an individual IFITM alone.

Studies on SARS-CoV-1 and recent papers and preprints on SARS-CoV-2 have shown a variety of phenotypes with different IFITMs on both viral entry and cell-to-cell fusion mediated by the spike protein (5, 19, 34). IFITM1 appears to block syncytium formation between infected and uninfected cells, and this is overcome by TMPRSS2 expression, which is consistent with our observations that in stably expressing cells, small effects of IFITM1 on SARS-CoV-2 entry in A549-ACE2 cells can be abolished similarly (35). Other data have implicated IFITM3 and demonstrated that it can be enhancing if its expression is restricted to the cell surface (19). Most of these experiments were performed in transiently transfected 293T cells with PLVs, and while the known determinants of IFITM3 function are required, whether transient overexpression faithfully represents the localization and potency of IFITM2 and IFITM3 natural expression is unclear. Furthermore, in mouse embryo fibroblasts, murine IFITM3 was shown to impart an IFN-regulated block to SARS-CoV-2 infection. However, it should be borne in mind that human IFITM2 and IFITM3 are more closely related to each other than either of them is to mouse IFITM3. Given the amount of positive selection that has occurred in the mammalian IFITM locus, species-specific differences in the spectrum of viruses restricted by mammalian IFITM orthologues should be expected (36).

Here, we find that stable ectopic expression of IFITM2 and to some extent IFITM1 restricts both the entry of PLVs and the replication of the SARS-CoV-2 virus itself in A549-ACE2 cells. The enhanced sensitivity of the PRRA mutant of SARS-CoV-2 and SARS-CoV-1 to IFITM2 is entirely consistent with their dependence on low-pH compartments for cathepsin cleavage. By restricting IFITM2 to the plasma membrane and the outside of clathrin-coated pits by abolishing AP2 interaction, we see enhancement effects similar to those seen by the Yount group with IFITM3 (34). Why this happens is not known, but given the effects that IFITMs have on membrane fluidity, this may be an indirect effect on the surface levels and distribution of entry cofactors at the plasma membrane. It also suggests why there may be an association of the rs12252-C polymorphism that expresses an N-terminally truncated IFITM3 with COVID-19 severity (37). Restriction by IFITM2 but not IFITM3 is surprising. This would suggest not only that IFITM2 localization is limited to later endosomes than IFITM3 but also that it may reside in distinct localizations at or near the plasma membrane dependent on its AP2-binding site.

In addition to examining the sensitivity of SARS-CoV-2 to individual IFITM proteins, we also showed that IFITM2 knockdown is sufficient to alleviate much of the antiviral effect of pretreating A549 cells with type I, but not type II, IFN. Studies from many groups have shown that while SARS-CoV-2 is a poor inducer of IFN responses in infected cells early in the replication cycle, it is highly sensitive to pretreatment of target cells by type I, II, and III IFNs (17, 18, 38). This suggests the potential for multiple ISGs to restrict SARS-CoV-2 replication and has raised the possibility of IFNs as possible treatments for COVID-19 (39). The role of IFNs in SARS-CoV-2 pathogenesis is complex. Genetic lesions in pattern recognition and IFN signaling as well as serum autoantibodies that neutralize type I IFNs are associated with risk of severe coronavirus disease 2019 (COVID-19) (40). However, dysregulated or delayed IFN responses driving systemic inflammation may underlie some of the pathology in COVID-19 (41). Understanding which aspects of the IFN response are antiviral against SARS-CoV-2 is thus of very high importance.

In A549-ACE2 cells, IFITM2 is more potently induced by IFN-β than IFN-γ, and its knockdown substantially reduces the sensitivity of the virus to IFN-β-induced restriction. The sequence similarity between IFITM2 and IFITM3 means that it is difficult to knock down one without affecting the other. The lack of IFITM3 restriction when expressed alone and its potent expression after both IFN-β and IFN-γ treatment would argue against IFITM3 playing the major role. However, given that IFITMs can interact with each other, we cannot rule out the possibility that IFITM1 or IFITM3 plays a role in potentiating IFITM2’s antiviral activity after IFN induction. The former is a distinct possibility, as IFITM2 knockdown fully rescues SARS-CoV-2 from IFN-β treatment in cells overexpressing TMPRSS2. Since we found that the minor restriction conferred by IFITM1 alone is abolished by TMPRSS2 expression, a plausible explanation is that more robust S2′ activation of SARS-CoV-2 spike at the cell surface overcomes IFITM1inhibition by saturating its activity (42). While it is surprising that IFN-β has no effect in these cells when IFITM2 is knocked down, we would caution against interpreting that IFITM2 is the only ISG targeting SARS-CoV-2 replication. The rapidity and burst size of SARS-CoV-2 replication in culture may render other relevant antiviral proteins difficult to measure. Furthermore, the virus encodes a number of antagonists of antiviral pathways (43). As shown clearly by the IFN-γ phenotype, expression of other ISGs or their differential regulation may make a given antiviral more or less potent. Of note, the IFN-γ-mediated inhibition of SARS-CoV-2 has been demonstrated to be in part mediated through the zinc-finger antiviral protein (ZAP) (38).

Despite the sensitivity of SARS-CoV-2 to IFITM2, deletion of the PRRA cleavage site in spike substantially potentiates its antiviral activity. In most cells in culture expressing low levels of TMPRSS2, furin cleavage is detrimental to entry, and in A549 cells, this can be rescued to mutant levels of entry by ectopic expression of TMPRSS2. In primary lung epithelial cells, however, the wild-type spike is clearly superior and outcompetes the mutant as well as being more transmissible in ferret models (5). Epithelial barrier tissues constitutively express a level of ISGs through the tonic activity of type I IFNs (44). Therefore, it is tempting to speculate that the selection pressure for maintaining this attribute in SARS-CoV-2 spike is in part to promote cell surface fusion in target cells that already express IFITM2. Interestingly, Peacock et al. have shown that an equivalent PRRA mutant virus can be rescued in lung epithelial cells by the antifungal drug amphotericin B, known to disrupt IFITM function (5). Addition of a partially active PRRA cleavage site is not sufficient to reduce the IFITM2 restriction of SARS-CoV-1 S to that of SARS-CoV-2, and thus, other determinants in spike are likely to modulate sensitivity.

In summary, we show that IFITM2 is a key antiviral protein targeting SARS-CoV-2 entry and its activity is modulated by the furin cleavage site in spike. These data therefore suggest that therapeutic strategies which upregulate IFITM2 in epithelial tissues or inhibit furin-mediated cleavage of spike may render the virus more sensitive to innate-immune mediated control.

## MATERIALS AND METHODS

### Cell lines and plasmids.

293T-17 (ATCC), A549-ACE2, A549-ACE2-TMPRSS2, Calu3 (ATCC), Vero E6, and A549-ACE2 cells expressing the individual IFITM proteins were cultured in Dulbecco’s modified Eagle medium (DMEM) (Gibco) with 10% fetal bovine serum (FBS) (Invitrogen) and 200 μg/ml gentamicin (Sigma) and incubated at 37°C and 5% CO_2_. Codon-optimized SARS-CoV-1 spike was synthesized by GeneArt, and codon-optimized SARS-CoV-2 spike and ACE2 were kindly provided by Nigel Temperton. Plasmid containing the TMPRSS2 gene was kindly provided by Caroline Goujon. The following mutants of spike or IFITMs were generated with a Q5 site-directed mutagenesis kit (E0554) following the manufacturer’s instructions: SARS-CoV-2 spike ΔPRRA (AGAAGCGTGGCCAGCCAG, GCTATTGGTCTGGGTCTGGTAG), SARS-CoV-1 spike PRRA (AGAGCCCGGAGCACCAGCCAGAAA, TCTAGGCAGCAGAGACACGGTGTG), IFITM2 Y19A (GCCTCCCAACgctGAGATGCTCAAGGAGGAG, TGGCCGCTGTTGACAGGA), and IFITM2 Y19F (GCCTCCCAACtttGAGATGCTCAAGGAG, TGGCCGCTGTTGACAGGA).

A549 stable cell lines expressing ACE2 (pMIGR1-puro), TMPRSS2 (IRES-neoWPRE), and IFITMs (pLHCX) were generated through transducing cells with lentiviral or retroviral vectors packaged with HIV Gag-Pol (8.91) or murine leukemia virus (MLV) Gag-Pol and vesicular stomatitis virus G protein (VSV-G). Cells were incubated with lentiviral or retroviral vectors for 4 h. Corresponding antibiotic selection was added 24 h after transduction.

### Production of PLVs and infection.

293T-17 cells were transfected with firefly luciferase-expressing vector (CSXW), HIV Gag-Pol (8.91), and spike at a ratio of 1.5:1:0.9 μg using 35 μl of PEI-MAX as previously described (45). Medium was changed 18 h later, and vectors were harvested through a 0.45-μm filter 48 h after transfection. Viral supernatant was then used to transduce each cell line of interest for 48 h, and readout was measured with a Promega Steady-Glo luciferase assay system (E2550).

### Passage and titration of SARS-CoV-2.

PHE England strain 02/2020 was propagated in Vero E6 cells, and titers were determined by plaque assay (46). Vero E6 cells were infected with serial dilutions of SARS-CoV-2 for 1 h. Subsequently, 2× overlay (DMEM, 2% FBS, and 0.1% agarose) medium was added, and infected cells were fixed and stained with crystal violet at room temperature 72 h later. Plaques were counted, and MOI was calculated for subsequent experiments.

### Infection with replication-competent SARS-CoV-2.

A549-ACE2 cells (1.5 × 10^5^) were infected for 1 h at 37°C with replication-competent SARS-CoV-2 at an MOI of 0.005. Medium was replaced, and cells were incubated for 48 h at 37°C. Forty-eight hours later, cells were harvest for RNA extraction or protein analysis, and the supernatant was used to infect Vero E6 cells to measure virus infectivity.

### Interferon assays.

Cells were treated with different doses of IFN-α (Invitrogen; 111001) IFN-β (PBL Assay Science; 11415-1), IFN-γ (Peprotech; 300-02), or IFN-λ (Peprotech; 300-02L) for 18 h prior to infection. Medium was changed for virus or PLVs the following day, and the infection was measured by Steady-Glo assay, qPCR, or N staining 48 h later.

### siRNA knockdown of IFITM2.

A549-ACE2 cells (1 × 10^5^) were reverse transfected using 20 pmol of nontargeting siRNA (Dharmacon catalog no. D-001206-13-20) or IFITM2 siRNA (Dharmacon catalog no. M-020103-02-0010) and 1 μl of RNAi Max (Invitrogen). Cells were incubated for 24 h prior to a second round of reverse transfection. Eight hours later, cells were treated with different doses of IFN-β or IFN-γ as described above.

Following 16 h of IFN treatment, cells were infected with replication-competent SARS-CoV-2 at an MOI of 0.005, as described above. Forty-eight hours after infection, cells were harvested for protein analysis, and the supernatant was used to measure virus infectivity by N staining.

### RT-qPCR.

RNA from infected cells was extracted using Qiagen RNeasy (Qiagen RNeasy minikit; 74106) following the manufacturer’s instructions. One microliter of each extracted RNA was used to performed one-step RT-qPCR using TaqMan fast virus one-step master mix (Invitrogen). The relative quantities of nucleocapsid (N) gene were measured using a SARS-CoV-2 (2019-nCoV) CDC qPCR probe assay (IDT DNA Technologies).

### SARS-CoV-2 N staining.

Vero E6 cells (2 × 10^4^) were infected for 1 h with 50 μl of undiluted or 1/10-diluted virus supernatant. Following infection, 50 μl of 2× overlay (DMEM, 2% FBS, and 0.1% agarose) medium was added to infected cells. Twenty-four hours later, cells were fixed using 4% paraformaldehyde for 30 min at room temperature. Fixed cells were permeabilized with 0.1% Triton for 15 min, blocked using 3% milk, and incubated for 45 min with anti-human anti-SARS-CoV-2 N (CR3009). Following incubation, cells were washed with 1× phosphate-buffered saline (PBS) and incubated with secondary antibody, goat anti-human IgG (Fc) peroxidase conjugate (Sigma A0170), for 45 min. Finally, the presence of N protein was determined using 1-Step Ultra TMB-ELISA substrate solution (Thermo Fisher).

### Drug assays.

Cells were pretreated with camostat mesylate (Sigma; SML0057), E64D (Sigma; E8640), or concanamycin (Cayman Chemicals; 80890-47-7) for 1 h at 37°C prior to transduction. Cells were then transduced with PLVs for 48 h, and infection was determined by luciferase activity.

### Influenza A virus multicycle replication assay.

Cells were infected with a NanoLuc luciferase-tagged influenza A virus (A/WSN/33), WSN-PASTN, a kind gift from Andrew Mehle (University of Wisconsin–Madison, Madison, WI, USA) (47). The inoculum was prepared in serum-free DMEM, and cells were inoculated at an MOI of 0.001 for 1 h at 37°C. After inoculation, cells were washed in PBS and grown in Opti-MEM (Gibco). NanoLuc expression was measured at 24 h postinfection using the Nano-Glo luciferase assay system (Promega) according to the manufacturer’s instructions.

### SDS-PAGE and Western blotting.

Cellular samples were lysed in reducing Laemmli buffer at 95°C for 10 min. Supernatant samples were centrifuged at a relative centrifugal force (RCF) of 18,000 through a 20% sucrose cushion for 1 h at 4°C prior to lysis in reducing Laemmli buffer. Samples were separated on 8 to 16% Mini-Protean TGX precast gels (Bio-Rad) and transferred onto nitrocellulose membranes. Membranes were blocked in milk prior to detection with the following antibodies: 1:1,000 rabbit anti-ACE2 (Abcam; Ab108209), 1:1,000 rabbit anti-TMPRSS2 (Abcam; Ab92323), 1:2,000 mouse anti-actin (Abcam; Ab6276), 1:5,000 rabbit anti-GAPDH (Abcam; Ab9485), 1:5,000 mouse anti-HSP90 (Genetex; Gtx109753), 1:50 mouse anti-HIV-1 p24Gag (48), 1:1,000 mouse anti-spike (Genetex; Gtx632604), 1:3,000 mouse anti-IFITM1 (Proteintech; 60074-1-Ig), 1:3,000 rabbit anti-IFITM2 (Proteintech; 12769-1AP), and 1:3,000 rabbit anti-IFITM3 (Proteintech; 11714-1-AP). Proteins were detected using Li-Cor and ImageQuant LAS 4000 cameras.
